# Modulation of Host Autophagy during Bacterial Infection: Sabotaging Host Munitions for Pathogen Nutrition

**DOI:** 10.3389/fimmu.2016.00081

**Published:** 2016-03-03

**Authors:** Pedro Escoll, Monica Rolando, Carmen Buchrieser

**Affiliations:** ^1^Institut Pasteur, Biologie des Bactéries Intracellulaires, Paris, France; ^2^CNRS UMR 3525, Paris, France

**Keywords:** autophagosome, xenophagy, autophagy modulators, intracellular bacteria, bacterial nutrition

## Autophagy is a Defense Mechanism Against Invading Pathogens

Cellular homeostasis requires the balanced regulation of anabolic and catabolic processes. While anabolic metabolism consumes energy to build up cellular components, catabolic processes break down organic matters in order to provide energy for the cell and its anabolic processes. Autophagy is a highly conserved and regulated catabolic process by which the eukaryotic cell degrades unnecessary, undesirable, or dysfunctional cellular components, including organelles (1–3). Autophagy is induced by a variety of extra- and intracellular stress stimuli, such as nutrient starvation, oxidative stress, or accumulation of damaged organelles or toxic protein aggregates. Initiation of autophagy first leads to the formation of cup-shaped structures known as phagophores that engulf the undesirable or damaged cellular components. Subsequent elongation of phagophores form double-membrane vesicles called autophagosomes, which deliver their cargo to lysosomes where the content is degraded and recycled (1–3). Autophagy plays a central role in quality control of organelles and proteins, and additionally is a key mechanism to maintain cellular energy levels and nutrient homeostasis during starvation, promoting the recycling and salvage of cellular nutrients. Furthermore, the cellular autophagic machinery is also used to remove invading intracellular pathogens, a process called xenophagy (1, 2). In this case, phagophores engulf invading microbes forming autophagosomes and steering them toward lysosomal degradation. Thus, xenophagy is an innate immune mechanism against bacterial infection that has been shown to be essential to restrict intracellular growth of many bacteria such as *Salmonella enterica* serovar Typhimurium (4), *Mycobacterium tuberculosis* (5, 6), *Listeria monocytogenes* (7), or Group A *Streptococcus* (8).

**Figure 1:**
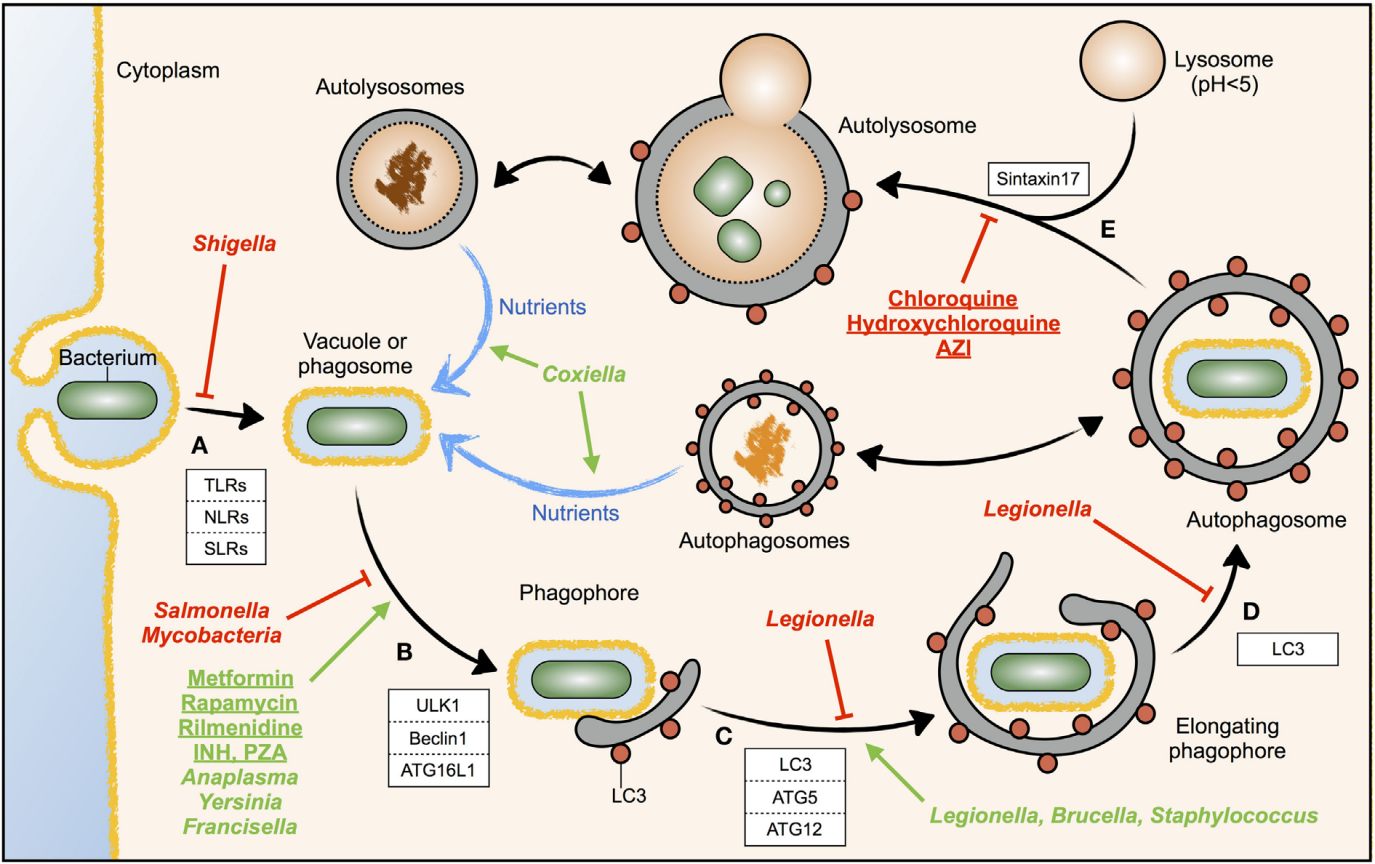
**Modulation of autophagy by drugs or intracellular bacteria**. The different steps of the autophagic response during bacterial invasion are shown. The host factors known to participate in each step are depicted in white boxes. **(A)** Invading bacteria are sensed by immune receptors; **(B)** vesicle nucleation induced by specialized autophagy proteins; **(C)** phagophore elongation; **(D)** autophagophore completion; **(E)** autophagosome maturation by fusion with lysosomes, forming autolysosomes. Drugs (underlined) or intracellular bacteria (*cursiva*) inducing autophagy are displayed in green, while those inhibiting autophagy are displayed in red. The different steps where bacteria or drugs act are pointed with green arrows (activation) or red T-bars (inhibition). Blue arrows indicate nutrient flow, while doubled-headed arrows indicate the possibility that the content of cellular autophagosomes and autolysosomes can be diverted to the phagosome and used by pathogenic bacteria as a source of nutrients.

Detection of bacterial components in the cytoplasm of mammalian cells induces autophagy via the activation of toll-like receptor 4 (TLR4) by bacterial lipopolysaccharide (LPS) and recognition of bacterial peptidoglycan by NOD1 and NOD2 (9, 10). TLR- and NOD-like receptor (NLR)-induced autophagy can be initiated during entry, uptake, or phagocytosis of bacteria by the host cell (10, 11), but bacteria can also be sensed by the Sequestosome-1-like receptors (SLRs) when they are already in the cytosol (1) (Figure 1A). In both cases, recruitment of autophagy proteins to the phagosome, such as the ULK1 complex, Beclin1, and ATG16L1, initiates membrane nucleation of the phagophore that will engulf the intracellular bacteria (10–12) (Figure 1B). ATG5–ATG12 associates with ATG16L1 and the ATG5–ATG12–ATG16L1 complex facilitates the addition of a phosphatidylethanolamine (PE) group to the carboxyl terminus of LC3, which function together with other factors to assemble, elongate, and allow the closure of nascent autophagosomes (1) (Figure 1C). In addition to this canonical mechanism of autophagy, phagosomes containing bacteria can recruit directly LC3, a process called LC3-associated phagocytosis (LAP). Upon delivery to phagosomes, LC3 promotes phagosome maturation and degradation of the content. Therefore, both LAP and canonical autophagy involve the enclosure of bacteria in an LC3-decorated compartment that is targeted for degradation by fusion with the lysosome (2). Membranes from the ER, the Golgi apparatus, the ER–mitochondria contact sites, or the plasma membrane contribute to the elongation of the double membrane of the phagophore in order to form the autophagosome (1) (Figure 1D). The attachment of syntaxin 17 to the autophagosomal membrane enables the fusion with lysosomes and represents the final maturation step of autophagosomes into autolysosomes (13) (Figure 1E), which normally leads to bacterial degradation in case of infection-induced autophagy (2).

## Pathogenic Intracellular Bacteria Subvert and Exploit the Autophagy Machinery of the Host

Xenophagy is a defense mechanism of the infected cell against invading bacteria, but intracellular pathogens have evolved mechanisms to inhibit or modulate the autophagy response of the host. For example, *M. tuberculosis* and *Salmonella* Typhimurium inhibit autophagy initiation signaling upstream autophagosome formation (14, 15), whereas *Shigella flexneri* evades autophagy recognition by masking the bacterial surface (16) (Figure 1, *cursive*, red).

In contrast to inhibition of autophagy, certain pathogenic intracellular bacteria induce autophagy and take advantage of it (17) (Figure 1, *cursive*, green). These bacteria show defective replication in autophagy-deficient cells, and treatment of host cells with autophagy activators promotes bacterial replication. This observation raises the question, why a pathogen would increase a host defense mechanism like autophagy? In uninfected cells, augmentation of the autophagy rate is used to increase the intracellular pool of basic nutrients, to build new cellular structures. During infection, some intracellular bacteria have developed mechanisms to hijack the autophagosomes and redirect the by-products of the autophagic degradation toward microbial replication rather than for the use by the host cell (18). In most cases, these bacteria actively induce autophagy but, at the same time, block autophagosome maturation and fusion with the lysosome. In this case, augmentation of autophagy, rather than promoting bacterial clearance, promotes the acquisition of nutrients by the invading bacteria (18). Thus, certain bacteria may sabotage the host defense mechanism elicited by autophagosomes to use the autophagic vesicles as nutrient source for microbial growth.

An example is *Anaplasma phagocytophilum* that uses a secreted effector, Ats-1, to promote autophagosome nucleation and stimulates its own growth by using the nutrients contained in the autophagosomes (19). Indeed, autophagy induction using rapamycin favors bacterial infection, while autophagy inhibition decreases *A. phagocytophilum* replication (20). Another example is *Yersinia pseudotuberculosis*, a Gram-negative bacterium that replicates intracellularly by establishing a specialized compartment, the *Yersinia*-containing vacuole (YCVs), which accumulates autophagy markers (21). The stimulation of autophagy with rapamycin increases the size of the YCVs and the numbers of replicative bacteria in the YCVs, whereas autophagy inhibition restricts bacterial survival, suggesting that autophagy promotes *Y. pseudotuberculosis* replication (21). *Yersinia pestis* also replicates within YCVs decorated with autophagosome markers (22). The authors suggested that autophagosomes may provide a source of membrane, along with late endosomes, for the expansion of the YCV into a spacious compartment (22). The same mechanism was described for *Coxiella burnetii*, the causative agent of Q fever. *Coxiella*-replicative vacuoles (CRVs) are decorated with the autophagy proteins LC3, Beclin1, and Rab24, and overexpression of LC3 or Beclin1 increases the number and size of the CRVs (23, 24). Similar to *A. phagocytophilum and Y. pseudotuberculosis*, autophagy induction increases *C. burnetii* replication, while inhibition of autophagy blocks *Coxiella* vacuole formation (23, 25). Also, *Francisella tularensis*, a highly virulent Gram-negative bacterium responsible for tularemia, avoids xenophagy while inducing autophagy (26). It was shown that autophagy-derived radiolabeled amino acids are transferred from host proteins to *F. tularensis*, a process that was reduced when host cells were treated with autophagy inhibitors (26).

Other bacteria also co-opt the autophagic machinery for their benefit, although a direct relationship of host autophagy and pathogen nutrition has not been shown. *Brucella abortus* that causes brucellosis in humans replicates in ER-derived *Brucella*-containing vacuoles (BCVs). BCVs hijack autophagosome initiation factors, such as ULK1 or Beclin1, and become autophagosome-like compartments (27). Depletion of ULK1 and Beclin1, as well as pharmacological inhibition of autophagy, readily reduced BCV formation, suggesting that autophagy promotes *B. abortus* infection (27). *Staphylococcus aureus* was also reported to be sequestered in LC3-positive autophagosomes that evade the fusion with lysosomes (28). *S. aureus* uses α-toxin to induce autophagy by an ATG5-dependent mechanism that also involves reduction of cellular cAMP levels (29). Infection of cells depleted of ATG5 show decreased bacterial replication, showing that autophagy is necessary for *S. aureus* replication *in vitro* (28).

Thus, different pathogenic bacteria seem to employ a common strategy to subvert the autophagy machinery as they not only target autophagy proteins to block xenophagy set up by the cell to resist infection but also exploit autophagy to promote their own replication. One well studied example for this dual strategy is the Gram-negative intracellular bacterium *Legionella pneumophila*. After phagocytosis, the causative agent of Legionnaires’ disease, forms a *Legionella*-containing vacuole (LCV) that recruits vesicles emerging from the endoplasmic reticulum (ER) and acquires autophagy markers like LC3, showing that LCVs rapidly become autophagosomes (30, 31). This process seems to be dependent on the T4SS bacterial effector LegA9, which promotes the recognition of the LCV by autophagy (32). Interestingly, inhibition of autophagy in permissive A/J mouse macrophages reduces *Legionella* survival at 2 h postinfection (30, 33), suggesting that routing the LCV to the autophagy pathway is beneficial for the bacteria. However, later, it has been shown that *L. pneumophila* is also restraining autophagy by secreting the specialized effectors, *Lp*SPL and RavZ, that inhibit, autophagosome formation and maturation, respectively (34, 35). The paradoxical existence in *Legionella* of bacterial effectors having opposite roles, on one hand, targeting the LCV to autophagy and, on the other hand, inhibiting autophagy may reflect the necessity for the bacteria to fine-tune host autophagy in a very balanced way. *Legionella* may need to target the LCV to autophagosomes, avoiding immediate killing (33), and at the same time, it needs to delay the maturation of the LCV-containing autophagosome into autolysosomes, gaining precious time for pathogen replication (30, 36).

## Autophagy Modulators in Infectious and Non-Infectious Diseases: Some Considerations

Autophagy modulators are of great interest for medical purposes (37), as it was suggested that metabolic, neurodegenerative, infectious, and oncology diseases can benefit from autophagy modulation (3).

One can hypothesize that drugs inducing autophagy could increase bacterial clearance in infected cells. This hypothesis is supported by the fact that antibiotics widely and extensively used against the intracellular bacterium *M. tuberculosis*, isoniazid (INH), and pyrazinamide (PZA), although able to kill the bacteria directly *in vitro* (38, 39), have been recently shown to induce autophagy in the host cell promoting mycobacterial clearance (40). Moreover, autophagy is required for effective antimycobacterial drug action *in vivo*, suggesting that pharmacological modulation of autophagy could be a successful strategy against infections by intracellular bacteria (40, 41). This point of view was corroborated by another recent report showing that treatment of cystic fibrosis patients with the antibiotic azithromycin (AZI) was associated with opportunistic mycobacterial infections. AZI was shown to prevent lysosomal acidification and thereby impaired autophagic degradation of mycobacteria (42), suggesting that chronic use of the drug may predispose to mycobacterial disease. Thus, these reports suggest that induction of autophagy with drugs, such as INH or PZA, could successfully treat mycobacterial infections, while inhibition of autophagy with drugs, such as AZI, may in turn facilitate mycobacterial infections.

Similar to mycobacteria, several molecules inducing autophagy have been recently shown to reduce *Salmonella* Typhimurium replication in HeLa cells (43, 44). This direct relationship between drugs, modulating autophagy and the outcome of bacterial infection, emphasizes the essential role of autophagy in the host response to intracellular bacteria and seems to support pharmacological modulation of host autophagy during infection. Unfortunately, as shown above, the situation seems more complex than the conclusion “increase of cellular autophagy favors bacterial clearance.”

The fact that autophagy inducers seem to be helpful in the treatment of *Mycobacteria* or *Salmonella* infections, but in turn might facilitate infections by *Anaplasma*, *Coxiella*, *Yersinia*, or *Francisella*, requires not only to be highly cautious in the use of autophagy modulators to treat infectious diseases but also to monitor the infectious risk during the use of autophagy modulators. Some autophagy modulators are already in use (Figure 1, underlined). Rapamycin, metformin, and rilmenidine, all autophagy inducers, are drugs approved and prescribed to prevent rejection of kidney transplants, to treat type 2 diabetes, and to treat hypertension, respectively (3, 37). In contrast, chloroquine and hydroxychloroquine, which are now under clinical trials as autophagy inhibitors for the treatment of certain resistant cancers, are drugs prescribed to treat malaria (3, 37). Moreover, hydroxychloroquine combined with doxycycline is currently used to treat *Coxiella-*induced chronic Q fever endocarditis (45). Some of these approved drugs might thus show a therapeutical benefit in case of infection.

In summary, the study of autophagy regulation during bacterial infection certainly shows the existence of a critical balance between a host-protective “immune-related” induction of autophagy (xenophagy) and a host-deleterious “metabolic-related” induction of autophagy by invading bacteria for nutritional theft of host energy resources. Results of clinical trials using autophagy modulators and a more profound understanding of the role of autophagy during infection are thus needed to correctly use autophagy modulators in the fight against infectious diseases.

## Author Contributions

All authors listed, have made substantial, direct and intellectual contribution to the work, and approved it for publication.

## Conflict of Interest Statement

The authors declare that the research was conducted in the absence of any commercial or financial relationships that could be construed as a potential conflict of interest.
